# The Inhibition of P-Selectin Reduced Severe Acute Lung Injury in Immunocompromised Mice

**DOI:** 10.1155/2020/8430465

**Published:** 2020-04-23

**Authors:** Yang Liu, Du Xiang, Fang Gao, Hanlin Yao, Qifa Ye, Yanfeng Wang

**Affiliations:** ^1^Zhongnan Hospital of Wuhan University; Institute of Hepatobiliary Diseases of Wuhan University; Transplant Center of Wuhan University, Hubei Key Laboratory of Medical Technology on Transplantation, Wuhan 430071, China; ^2^Binzhou People's Hospital Health Management Center, Binzhou 256600, Shandong Province, China; ^3^Research Center of National Health Ministry on Transplantation Medicine Engineering and Technology, The 3rd Xiangya Hospital of Central South University, Changsha 410000, China

## Abstract

In an immunocompetent host, excess infiltration of immune cells in the lung is a key factor in infection-induced severe acute lung injury. Kidney transplant patients are immunocompromised by the use of immunosuppressive drugs. Immune cell infiltration in the lung in a renal transplant recipient suffering from pulmonary infection is significantly less than that in an immunocompetent host; however, the extent of lung injury in renal transplant patients is more serious than that in immunocompetent hosts. Therefore, we explored the role of platelet activation in a Klebsiella pneumoniae-induced lung injury model with P-selectin gene knockout mice or wild-type mice. Our study suggested that the inhibition of platelets reduced severe acute lung injury and increased survival after acute lung infection in mice. In addition, P-selectin expression on the surface of platelets in mice increased after administration of immunosuppressive drugs, and the extent of lung injury induced by infection decreased in P-selectin gene knockout mice. In conclusion, p-selectin plays a key role in severe acute lung injury in immunocompromised mice by reducing platelet activation and inflammatory processes.

## 1. Introduction

Renal transplantation is the best treatment for end-stage renal disease. Due to the use of immunosuppressive drugs, the immunity of kidney transplant recipients is obviously impaired, which easily induces postoperative infection, especially pulmonary infection [1, 2]. Approximately 10-20% of patients suffer from pulmonary infection after kidney transplantation [3]. Severe acute lung injury caused by infection is the main cause of early death [4]. At present, there is no effective treatment for severe acute lung injury.

When the body is infected, the immune system is activated and defends against infection through the following processes. First, macrophages in the alveoli eradicate pathogens, produce chemokines, and induce circulating polymorphonuclear leukocytes (PMNs) to accumulate in pulmonary microvessels [5]. Second, the binding of selectin and its ligand mediates the interaction between PMNs, platelets and vascular endothelial cells, which induces the PMNs to adhere to the vascular intima [5]. Third, activated PMNs migrate through the blood vessel wall to the lung tissue, produce inflammatory mediators, and attract more immune cells to aggregate in the lung; moreover, activated PMNs release active substances to eradicate pathogens [6]. Previous studies have suggested that excessive PMN infiltration in the lung is a key factor leading to severe acute lung injury [7–10]. However, continuous use of immunosuppressive drugs after renal transplantation reduces the immunity of patients. When pulmonary infection occurs, PMN infiltration in the lung in a renal transplant recipient is significantly less than that in an immunocompetent host; however, the extent of lung injury in renal transplant patients is more serious than that in immunocompetent hosts. Therefore, we hypothesized that other factors play an important role in severe acute lung injury induced by pulmonary infection after renal transplantation.

Numerous studies have shown that platelets are related to the inflammation [11–13]. Platelets participate in inflammation and release inflammatory factors to increase vascular permeability. Furthermore, platelets participate in inflammation by mediating PMN infiltration in the lung [14–17]. We hypothesized that immunosuppressive drugs significantly reduce PMN infiltration in the lung after renal transplantation, but platelets induce PMNs to adhere to pulmonary vascular endothelial cells, aggregate and activate in the lung, and release a large number of active factors, leading to severe acute lung injury.

P-selectin, also called granule membrane protein 140, antigen CD62, or platelet activation dependent granule-external membrane protein (PADGEM), is a 140 kD adhesion molecule that mediates the interaction of stimulated endothelial cells or platelets to leukocytes in the vascular surface [18]. Mayadas et.al confirmed that the combination of P-selectin and its ligand PSGL-1 mediates the adhesion of platelets to vascular endothelial cells and promotes platelet release and aggregation [19]. Moreover, the adhesion of platelets to the vascular endothelium releases platelet activating factor and other inflammatory mediators, resulting in increased permeability of the air-blood barrier [20]. Therefore, p-selectin may play an important role in lung injury after kidney transplantation.

At present, the role of platelets in severe acute lung injury is incompletely understood. In the present study, we aimed to explore the effects of platelet P-selectin on severe acute lung injury in immunocompromised mice.

## 2. Materials and Methods

### 2.1. Animals

Wild-type male C57BL/6 mice (20-25 g) were purchased from the Center for Animal Experiments of Wuhan University (Wuhan, China). P-selectin gene knockout mice were purchased from Jackson Laboratory (Bar Harbor, ME, USA). The mice were housed in a temperature- and humidity-controlled (40%) animal room at 25°C with a 12 h light/dark cycle and free access to food and water. All experimental procedures were approved by the ethical committee of Wuhan University. The mice were anesthetized intraperitoneally with 40 mg/kg sodium pentobarbital. Wild-type male C57BL/6 mice were used to establish the immunocompromised host (ICH) model and Klebsiella pneumoniae-induced lung injury (KPN) model. The mice were randomly assigned to the control, ICH, KPN, KPN + ICH, and KPN + ICH + clopidogrel (Clop) groups. The KPN + ICH + clopidogrel group (clopidogrel, 1.25 mg/kg body weight, dissolved in normal saline) was intraperitoneally administered once a day for three days prior to KPN modeling, and an equal volume of saline was intraperitoneally administered at the same frequency for the KPN + ICH group.

### 2.2. Immunocompromised Host (ICH) Model

To establish the animal model of ICH, eighteen C57BL/6 mice were divided into three groups (6 mice in each group): the control group, FK506 (tacrolimus) group and FK506 + DXM (dexamethasone) group. FK506 group mice received daily intraperitoneal injections of tacrolimus (0.3 mg/kg/d; Astellas Ireland Co., Ltd) for seven consecutive days. FK506 + DXM group mice received daily intraperitoneal injections of tacrolimus (0.3 mg/kg/d) and dexamethasone (50 mg/kg/d; Huazhong Pharmaceutical Co., Ltd) for seven consecutive days. Mice receiving normal saline injection were used as the control group. Seven days later, the mice were anesthetized and sacrificed, and blood, thymus tissues and spleen tissues were collected.

### 2.3. Klebsiella Pneumoniae Induced-Lung Injury (KPN) Model

The mice were intratracheally challenged with Klebsiella pneumoniae (0.05 ml; Cat. no. CMCC46114; National Center for Medical Culture Collections) at concentrations of 2 × 10^8^ CFU/ml, 6 × 10^8^ CFU/ml, 2 × 10^9^ CFU/ml or 6 × 10^9^ CFU/ml. Twenty-four hours after challenge with Klebsiella pneumoniae, the mice were anesthetized and sacrificed by inferior vena cava puncture and exsanguination. Blood and lung tissue samples were collected.

### 2.4. Hematoxylin and Eosin (H&E) Staining

Sections (4 − *μ*m thickness)were serially cut to perform the morphometric analysis of lung tissues. These sections were stained with hematoxylin for 15 min and eosin for 5 min at room temperature to perform histological analysis. Histological examination was performed under a light microscope (magnification: ×400; Olympus BX43; Olympus Corporation). In each tissue sample, five random areas were scored, and the mean value was calculated by the modified scoring system described by Hasan et.al [21] and XiaoLi Wang et.al [22]. The histology score was the sum of the following four parameters: size of alveolar spaces, thickness of alveolar septa, alveolar fibrin deposition and neutrophil infiltration (0: absent and appears normal; 1: light; 2: moderate; 3: strong; and 4: intense; total score is 16).

### 2.5. Wet/Dry Weight Ratio of the Lung

The change in the ratio of wet/dry weight was used as an indicator of lung edema formation. At 24 hours after Klebsiella pneumoniae or normal saline administration, the mice were anesthetized and sacrificed. Blood and lung tissue samples were collected. Gauze was used to dry the blood cells and water on the surface of the lung tissue. Then, the lung tissues were weighed and dried in an oven at 65°C for 24 h to obtain the lung wet/dry (W/D) ratio.

### 2.6. Systemic Platelet and Leukocyte Counts

Blood samples were collected from the mice 24 hours after Klebsiella pneumoniae or normal saline administration. Platelet and leukocyte counts were performed in ethylenediaminetetraacetic acid (EDTA)-anticoagulated blood using a hematology analyzer (Sysmex XN-350; Sysmex Corporation, Kobe, Japan).

### 2.7. Elisa

Serum samples were warmed to room temperature. Serum levels of P-selectin (Cat. no. SEA569Mu; CLOUD-CLONE CORP), TNF-*α* (Cat. no. 88-7324; Thermo Fisher Scientific), IL-6 (Cat. no. 88-7064; Thermo Fisher Scientific) and TAX2 (Cat. no. E-EL-0057c; Elabscience) were measured by ELISA kits according to the manufacturer's protocols.

### 2.8. Immunofluorescence

The lung tissues were fixed in 4% paraformaldehyde at 4°C overnight. The lung tissue of each group was serially sliced into 4 − *μ*mthick slices and then incubated with 5% goat serum (Beyotime Institute of Biotechnology) at 22°C for 1 h. Lung tissues from each group were sliced into 2 sections, and each sample was incubated with rabbit anti-CD41 primary antibodies (Cat. no., ab63983; both 1 : 100; Abcam, Inc.) at 4°C overnight and subsequently with fluorescein-conjugated mouse anti-rabbit IgG (Cat. no. GB25303; Servicebio, Inc.) for 1 h at room temperature. Following staining with DAPI (Cat. no. G1012; Servicebio, Inc.) for 5 min at room temperature, the samples were imaged using a wide-field fluorescence microscope (Olympus X-cite 120; Olympus Corporation; magnification: ×200).

### 2.9. Reverse Transcription Quantitative Polymerase Chain Reaction (RT-qPCR)

Total RNA was extracted from mouse lung tissues using TRIzol reagent (Invitrogen; Thermo Fisher Scientific, Inc.) according to the manufacturer's protocol. RNA was detected under a UV lamp after formaldehyde-modified agarose gel electrophoresis for 10 min (stained with ethidium bromide, buffered with 1× MOPS, and applied with a constant voltage of 5 V/cm) [23]. cDNA was synthesized according to the Avian Myeloblastosis Virus Reverse Transcriptase Protocol (Promega Corporation) and purified by the PAGE method. GAPDH mRNA was used as the loading control to ensure uniform loading of all RNA samples. The amplification conditions were 95°C for 10 min, followed by 40 cycles of 15 sec at 95°C and 1 min at 60°C. The transcription levels of target genes (Table 1) in all samples were compared with the internal reference gene GAPDH and were analyzed by the 2 - Cq method [24].

### 2.10. Statistical Analysis

Statistical analysis was performed using SPSS software (version 22.0; SPSS, Inc.). All data are presented as the mean ± standard deviation and were analyzed using Student's t-tests. All experiments were repeated in triplicate, and *P* < 0.05 was considered to indicate a statistically significant difference.

## 3. Results

### 3.1. Establishment of an Immunocompromised Mouse Model

**Figure 1:**
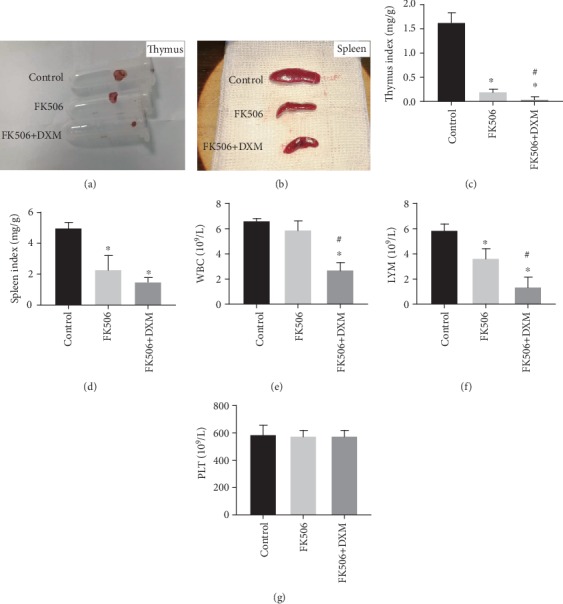
Establishment of immunocompromised mice model. (a) the thymus of mice; (b) the spleen of mice; (c) the thymus index of mice; (d) the spleen index of mice; (e–g) peripheral blood circulation leukocytes, lymphocytes and platelet counts. Values are expressed as mean ± SEM. ∗*P* < 0.05 vs control; ^#^*P* < 0.05 vs FK506 group (six mice per group).

Tacrolimus and dexamethasone are two immunosuppressive drugs used by patients after organ transplantation. Tacrolimus and dexamethasone were used to establish immunocompromised mouse models. Mice in the control group, FK506 group and FK506 + DXM group were intraperitoneally injected with normal saline, tacrolimus or tacrolimus plus dexamethasone, respectively, for seven consecutive days. During this period, there was no death in the three groups of mice. The thymus of the control group was plump and moist, the thymus of the FK506 group was atrophied, and the thymus of the FK506 + DXM group was severely atrophied or even disappeared (Figure 1(a)). Compared with the spleen sizes in the control group, the spleen sizes in the FK506 group decreased, and atrophy of the spleens in the FK506 + DXM group was more obvious (Figure 1(b)). Then, we calculated the thymus index and spleen index of the three groups of mice, which suggested that the thymus and spleen sizes in the FK506 group and FK506 + DXM group were markedly atrophied compared with those of the control group (*P* < 0.05). Furthermore, the thymus and spleen sizes in the FK506 + DXM group were smaller than those in the FK506 group (*P* < 0.05; Figure 1(c)–1(d)).

We counted white blood cells (WBCs) and platelets in the three groups of mice using a hematology analyzer. The results indicated that the number of circulating WBCs in the FK506 + DXM group was markedly decreased compared with that in the control group, but there was no significant difference between the control and FK506 groups (*P* < 0.05; Figure 1(e)). Moreover, we found that the inhibitory effect of the combination of tacrolimus and dexamethasone on circulating lymphocytes was better than that of tacrolimus alone (*P* < 0.05; Figure 1(f)). However, tacrolimus and dexamethasone had no significant effect on the numbers of circulating cells in mice (*P* > 0.05; Figure 1(g)). Tacrolimus and dexamethasone had strong immunosuppressive effects on the mice, and the combination of tacrolimus and dexamethasone was superior to that of tacrolimus alone.

### 3.2. Establishment of a Lung Injury Model in Immunocompromised Mice

The immunocompromised mice were inoculated with Klebsiella pneumoniae at four different concentrations (2 × 10^8^ CFU/ml, 6 × 10^8^ CFU/ml, 2 × 10^9^ CFU/ml or 6 × 10^9^ CFU/ml). Four to six hours after inoculation with Klebsiella pneumoniae mice in the ICH group manifested symptoms such as dispiritedness, polypnea, bleeding around the nose and mouth, and a reduced frequency of eating and drinking water. Mice in the 2 × 10^8^ CFU/ml and 6 × 10^8^ CFU/ml groups survived 24 hours after inoculation with Klebsiella pneumoniae. However, sixty percent of the mice in the 2 × 10^9^ CFU/ml group and eighty percent of the mice in the 6 × 10^9^ CFU/ml group that were inoculated with Klebsiella pneumoniae died before the scheduled test time. Therefore, high inoculation concentrations of Klebsiella pneumoniae caused death in the mice.

**Figure 2:**
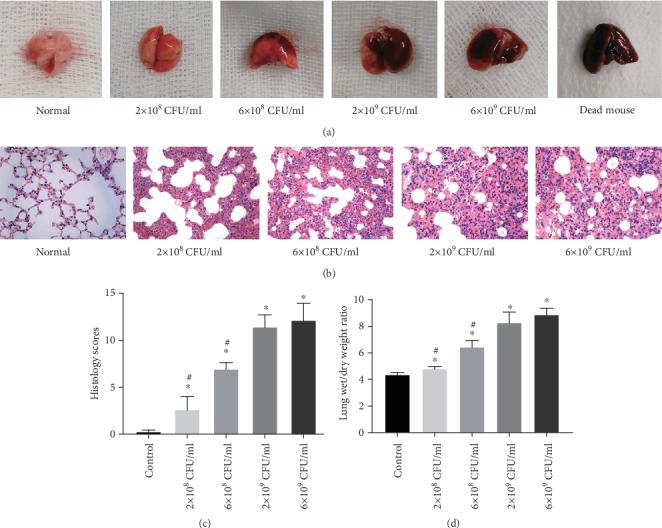
Establishment of lung injury model in immunocompromised mice. (a) The lung tissues of mice; (b) representative HE sections of the lung tissues (magnification, ×400); (c) lung histology scores; (d) the Wet/Dry weight ratio of lung tissues. Values are expressed as mean ± SEM.∗*P* < 0.05 vs control; ^#^*P* < 0.05 vs 2 × 10^9^ CFU/ml group (six mice per group).

In the control group, the lung surfaces of the mice were smooth and slightly white. After the inoculation with Klebsiella pneumoniae, pulmonary congestion and edema were observed on the surface of the lung, and some punctate or flaky hemorrhage was observed under the capsule. Furthermore, the extent of pulmonary hemorrhage and edema increased with the concentration of inoculated bacteria (Figure 2(a)). Then, HE staining was performed on the lung tissues (Figure 2(b)). The structure of the lung tissues in the control group was clear and complete. After inoculation with Klebsiella pneumoniae, the lungs showed various degrees of inflammatory reactions, including exudation and inflammatory cell infiltration in the bronchi, surrounding bronchi and alveoli, pulmonary interstitial edema, and capillary congestion (Figure 2(b)). As shown in Figure 2(c), histology scores were significantly higher in the KPN + ICH groups than in the control group. Moreover, the histological score of the lung increased with increasing concentrations of Klebsiella pneumoniae inoculation in the mice. However, there was no significant difference between the 2 × 10^9^ CFU/ml and 6 × 10^9^ CFU/ml groups (*P* < 0.05; Figure 2(c)).

To evaluate the extent of pulmonary edema in each group, the W/D weight ratio of lung tissues was determined. Consistent with the histological analysis, the lung W/D weight ratios in the 2 × 10^9^ CFU/ml and 6 × 10^9^ CFU/ml groups were significantly higher than those of the 2 × 10^8^ CFU/ml and 6 × 10^8^ CFU/ml groups (*P* < 0.05; Figure 2(d)). However, there was no significant difference between the 2 × 10^9^ CFU/ml and 6 × 10^9^ CFU/ml groups. In general, these results suggest that high concentrations of inoculated Klebsiella pneumoniae induce severe acute lung injury. Therefore, 2 × 10^9^ CFU/ml Klebsiella pneumoniae was used for subsequent experiments.

### 3.3. Treatment with Immunosuppressive Drugs Exacerbates Severe Acute Lung Injury in Mice

**Figure 3:**
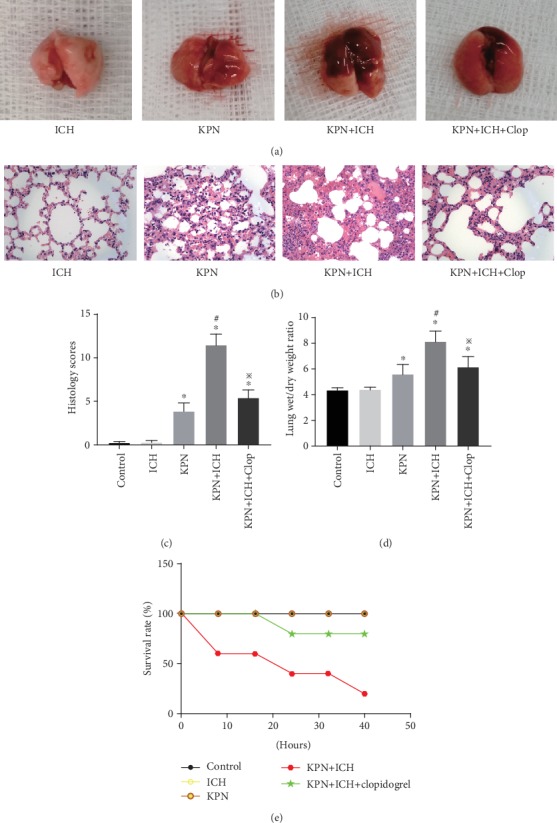
Effect of Clopidogrel pretreatment on lung injury mice. (a) The lung tissues of mice; (b) representative HE sections of the lung tissues (magnification, ×400); (c) lung histology scores; (d) The Wet/Dry weight ratio of lung tissues; (e) the survival curve of mice. Values are expressed as mean ± SEM. ∗*P* < 0.05 vs control; ^#^*P* < 0.05 vs KPN group; ^※^*P* < 0.05 vs KPN + ICH group (five mice per group).

Normal and ICH mice were inoculated with 2 × 10^9^ CFU/ml Klebsiella pneumoniae. The extent of lung injury was more severe in the KPN + ICH group than in the KPN group. After inoculation with Klebsiella pneumoniae, there were multiple patchy hemorrhages under the lung capsule in ICH mice, with obvious pulmonary congestion and edema (Figure 3(a)–3(b)). Likewise, lung histology scores and the W/D weight ratio were significantly higher in the KPN + ICH group than in the KPN group (Figure 3(c)–3(d)). Therefore, as shown in Figure 3(e), the survival rate of mice in the ICH group was significantly higher than that of KPN mice within 40 hours. These data indicate that the use of immunosuppressive drugs in the KPN + ICH group exacerbates severe acute lung injury caused by Klebsiella pneumoniae.

**Figure 4:**
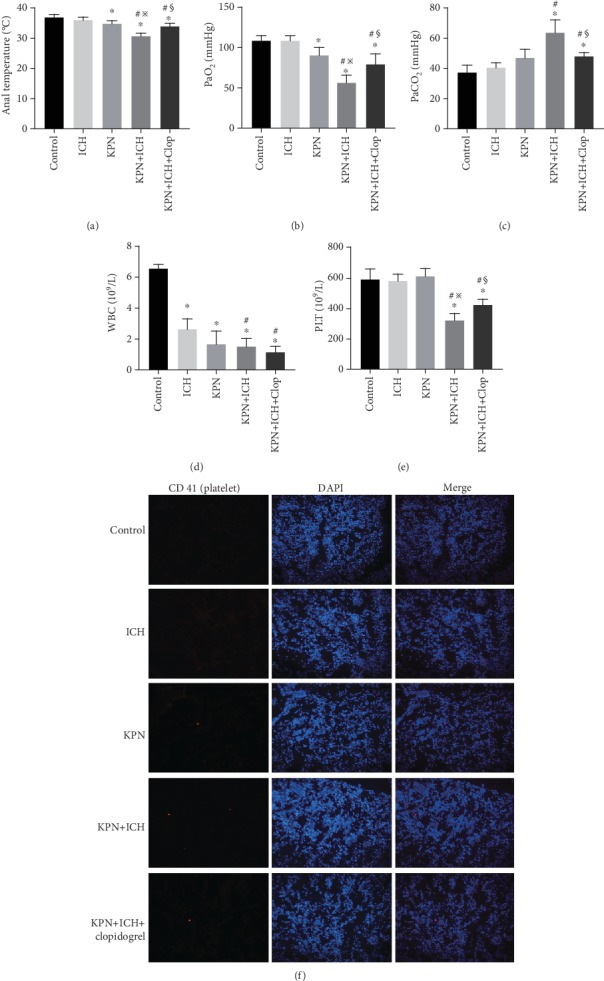
Clopidogrel alleviate acute lung injury and reduce platelet aggregation in lung tissues in mice. (a) Anal temperatures of mice; (b–c) Arterial oxygen partial pressure and carbon dioxide partial pressure of mice; (d–e) peripheral blood circulation leukocyte and platelet counts; (f) immunofluorescence sections of mice lung tissue. Values are expressed as mean ± SEM. ∗*P* < 0.05 vs control; ^#^*P* < 0.05 vs ICH group; ^※^*P* < 0.05 vs KPN group; ^§^*P* < 0.05 vs KPN + ICH group (five mice per group).

The anal temperatures and arterial partial pressure of oxygen (PaO2) decreased and the arterial partial pressure of carbon dioxide (PaCO2) increased significantly in the KPN and KPN + ICH groups compared with those in the control and ICH groups. Compared with KPN group mice, KPN + ICH group mice had lower anal temperatures and PaO2 and higher PaCO2. In addition, severe acute lung injury in mice induced with Klebsiella pneumoniae reduced the numbers of circulating WBCs (Figure 4(d)). Interestingly, Klebsiella pneumoniae-induced severe acute lung injury in ICH mice decreased the number of circulating platelets (*P* < 0.05; Figure 4(e)). However, thrombocytopenia did not occur in the severe acute lung injury model in normal mice in the KPN group (Figure 4(e)).

We stained lung tissues with antibodies against CD41 to detect platelet aggregation in lung tissues. In the control and ICH groups, the lung tissues did not show platelet aggregation. However, platelet aggregation was observed in Klebsiella pneumoniae-inoculated mice. As expected, there were increased platelet aggregates in the lung tissues of the KPN + ICH group mice (Figure 4(f)). These results indicate that the use of immunosuppressive drugs exacerbate severe acute lung injury, decrease the numbers of circulating platelets and increase the formation of platelet aggregates in the lung during pulmonary infection.

### 3.4. Blocking Platelet Aggregation with Clopidogrel Alleviates Severe Acute Lung Injury and Reduces Platelet Aggregation in Lung Tissues in Mice

Clopidogrel is a platelet aggregation inhibitor that selectively inhibits the binding of ADP and platelet receptors and inhibits activation of the glycoprotein GPIIb/IIIa complex on platelets [25]. Mice were intraperitoneally injected with 1.25 mg/kg clopidogrel (Salubris; China) for three consecutive days before inoculation with Klebsiella pneumoniae. Clopidogrel pretreatment attenuated Klebsiella pneumoniae-induced histological injury and the extent of lung edema in the KPN + ICH + Clop group compared to that of the KPN + ICH group (Figure 3(a)–3(d)). Furthermore, clopidogrel dramatically improved the survival rate of Klebsiella pneumoniae-inoculated ICH mice (Figure 3(e)). Systemic infection and lung ventilation disorder in KPN + ICH group mice were alleviated by clopidogrel (Figure 4(a)–4(c)). As expected, clopidogrel pretreatment increased the numbers of circulating platelets and reduced the formation of platelet aggregates in the lungs of KPN + ICH group mice (Figure 4(e)–4(f)). These data indicate that clopidogrel plays a positive role in protecting against severe acute lung injury and mitigating induced thrombocytopenia.

### 3.5. Blocking Platelet Aggregation by Clopidogrel Reduces the Levels of TNF-*α* and IL-6

**Figure 5:**
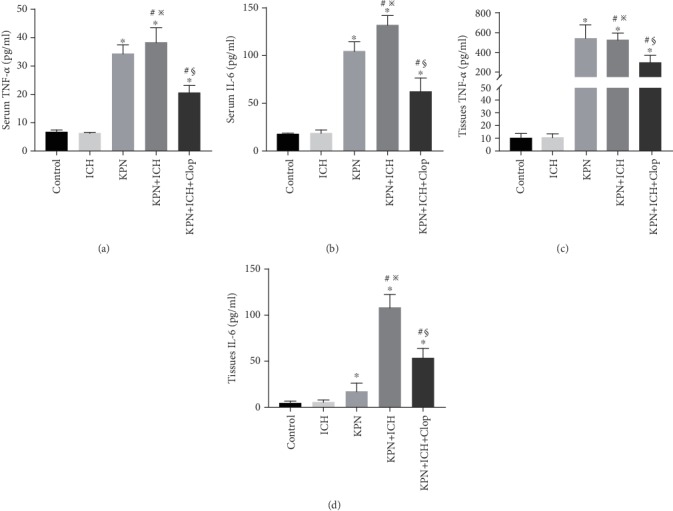
The levels of TNF-*α* and IL-6 in mice. (a–b) The expression of TNF-*α* and IL-6 in serum; (c–d) the expression of TNF-*α* and IL-6 in lung tissues. Values are expressed as mean ± SEM. ∗*P* < 0.05 vs control; ^#^*P* < 0.05 vs ICH group; ^※^*P* < 0.05 vs KPN group; ^§^*P* < 0.05 vs KPN + ICH group (five mice per group).

TNF-*α* and IL-6 are two proinflammatory cytokines that are indicators of inflammation [26, 27]. We detected TNF-*α* and IL-6 expression levels in serum and lung tissue by ELISA. As shown in Figure 5, the TNF-*α* and IL-6 levels in the KPN and KPN + ICH groups were higher than those in the control and ICH groups (*P* < 0.05). Furthermore, pretreatment with clopidogrel significantly reduced TNF-*α* and IL-6 expression levels in the KPN and KPN + ICH groups (*P* < 0.05). These data confirmed that platelet blockade induced by clopidogrel pretreatment significantly reduces the extent of inflammation in severe acute lung injury mice.

### 3.6. Knockout of P-Selectin Alleviates Severe Acute Lung Injury in ICH Mice

**Figure 6:**
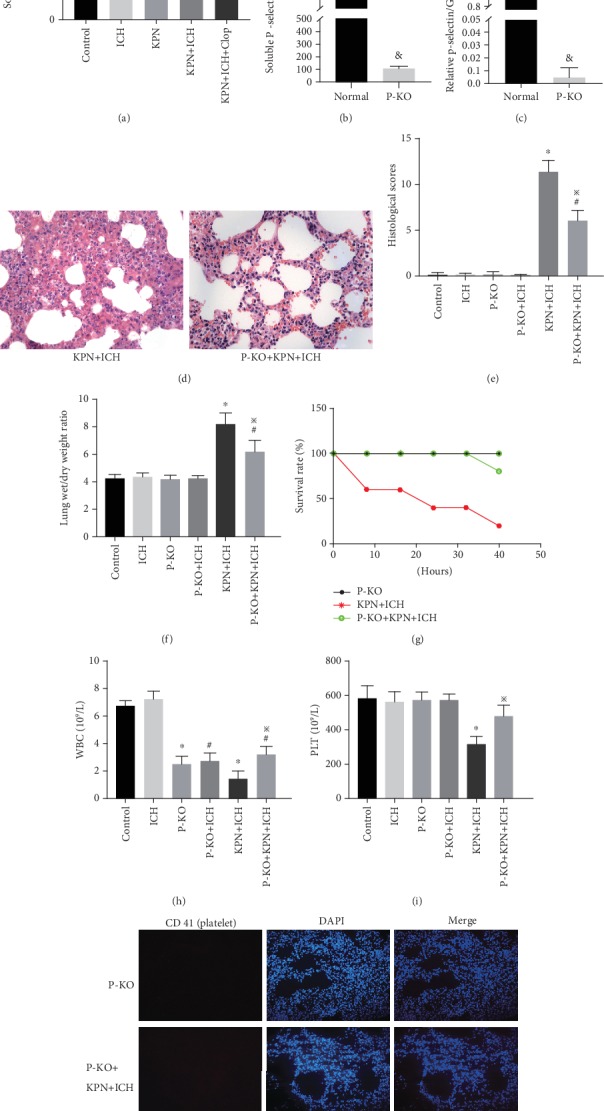
Knockout of p-selectin alleviate acute lung injury in ICH mice. (a) The expression of serum P-selectin in mice; (b–c) the expression of serum P-selectin in P-selectin knockout mice detected by Elisa assay and RT-PCR; (d) representative HE sections of the lung tissues (magnification, ×400); (e) lung histology scores; (f) The Wet/Dry weight ratio of lung tissues; (g) the survival curve of mice; (h–i) peripheral blood circulation leukocyte and platelet counts; (j) immunofluorescence sections of mice lung tissue. Values are expressed as mean ± SEM. ∗*P* < 0.05 vs control; ^&^*P* < 0.05 vs Normal group; ^#^*P* < 0.05 vs P-KO group; ^※^*P* < 0.05 vs KPN + ICH group(five mice per group).

P-selectin mediates the rolling of neutrophils and lymphocytes on platelets or activated endothelial cells and is involved in clotting, thrombosis and inflammation [28]. We found that P-selectin expression in mouse serum and lung tissues increased after administration of immunosuppressive drugs or inoculation with Klebsiella pneumoniae (*P* < 0.05; Figure 6(a)). The P-selectin expression in serum and lung tissues of KPN pneumoniae-inoculated ICH mice in the KPN + ICH group was higher than those in the KPN and ICH groups (*P* < 0.05; Figure 6(a)). Then, the effect of P-selectin on severe acute lung injury induced by Klebsiella pneumoniae in immunocompromised mice was evaluated in subsequent experiments. Compared with wild-type mice, p-selectin knockout mice showed low expression in serum and lung tissues (*P* ≤ 0.001; Figure 6(b)–6(c)). Likewise, P-selectin knockout mice were used to establish animal models of immunosuppression and severe acute lung injury. The results confirm that P-selectin knockout attenuated Klebsiella pneumoniae-induced severe acute lung injury and improved the survival rate of ICH mice that were inoculated with Klebsiella pneumoniae (Figure 6(d)–6(g)). Furthermore, P-selectin knockout increased the numbers of circulating WBCs and platelets and reduced the formation of platelet aggregates in the lungs of KPN + ICH group mice (Figure 6(h)–6(j)). These data suggest that P-selectin knockout alleviates Klebsiella pneumoniae-induced severe acute lung injury in ICH mice.

## 4. Discussion

With the wide development of organ transplantation and the wide clinical application of immunosuppressive drugs, the number of immunocompromised hosts is increasing. Refractory respiratory infection caused by immunosuppressive drugs is the main cause of death in ICHs [29]. Immunosuppressive drugs, including tacrolimus and glucocorticoids, are widely used in the immunotherapy of organ transplantation, chemoradiotherapy and immune-related diseases, and recipients often develop immune impairment characterized by leukopenia [30].

At present, many studies on ICHs have established animal models to simulate immunocompromised patients [31–33]. In our experiment, we used tacrolimus and dexamethasone to establish the ICH model in C57BL/6 mice. The ICH mouse model showed spleen atrophy, atrophy or even disappearance of the thymus, and significantly decreased peripheral blood leukocytes (*P* < 0.05).

Klebsiella pneumoniae is widely distributed in nature and is an important pathogen associated with acquired pneumonia in hospitals. In recent years, the incidence of Klebsiella pneumoniae has been on the rise [34, 35]. Kidney transplant patients are typical immunocompromised hosts. The continuous use of high-dose immunosuppressive drugs leads to a reduction in immune cells, including PMNs. Pulmonary infiltration of PMNs is significantly reduced compared with that of normal host infection. However, the extent of acute lung injury is more serious in ICHs than in normal hosts. Our study suggested that severe acute lung injury caused by pulmonary infection in ICH mice is related to thrombocytopenia, and the extent of thrombocytopenia is related to the extent of lung injury. Moreover, the extent of lung injury in C57 wild-type mice that were treated with clopidogrel was less than that in the control group. Therefore, we believe that platelets play an important role in severe acute lung injury after renal transplantation.

Numerous studies have confirmed that platelets play not only a leading role in coagulation and thrombosis but also a key role in inflammation [36–38]. Platelets in the blood mainly exert anti-infection effects through the following mechanisms: ① After inflammatory stimulation, platelet P-selectin is transferred from the cytoplasm to the surface of the cell membrane, inducing adhesion of PMNs to endometrial cells and promoting the formation of neutrophil extracellular traps [39, 40]. ② Activated platelets produce microparticles and participate in inflammatory reactions [41]. ③ Platelets contain alpha granules, dense granules, lysosomes and other particles. After platelet activation, various mediators are released, including coagulation factors, vasoactive substances and inflammatory mediators. These molecules have a dramatic impact on the permeability of blood vessels [42–44].④ Platelets synthesize molecules such as TNF-*α* and IL-6 under infection conditions and participate in the inflammatory response. Increasing evidence supports the important role of platelets in severe acute lung injury [45–47].

Graff J. et.al suggested that platelets are easily activated in transplant patients after immunosuppressive therapy [46]. Our study confirmed that the continuous use of high-dose immunosuppressive drugs in mice increases the expression of P-selectin on the platelet surface. Mayadas et.al. showed that the binding of P-selectin and its ligand PSGL-1 mediates the adhesion of platelets to vascular endothelial cells and promotes platelet release and aggregation [47]. Immunosuppressive drugs promote the adhesion of platelets to vascular endothelial cells [48]. Platelets adhere to the vascular endothelium and release some bioactive molecules, resulting in increased permeability of the air-blood barrier.

In our study, p-selectin gene knockout and wild-type mice were selected to establish immunocompromised animal models, and Klebsiella pneumoniae was inoculated into airways to establish an animal model of severe acute lung injury induced by pulmonary infection. Finally, we found that the extent of acute lung injury in p-selectin knockout mice was lower than that in wild-type mice. These results confirmed that p-selectin plays an important role in severe acute lung injury in immunocompromised hosts.

The present study demonstrated that blocking platelet aggregation with clopidogrel reduced severe acute lung injury in mice; however, clopidogrel exerts both platelet-dependent and platelet-independent anti-inflammatory effects [49]. The platelet-independent anti-inflammatory effect of clopidogrel also has a certain effect on severe acute lung injury in mice. Also, platelet aggregation in the lung was not observed in real time. It is necessary to use in vivo fluorescence microscopy imaging technology in subsequent experiments. Platelet lung aggregation in mice with severe acute lung injury can be observed in real time, and we can better understand the effects of platelets and p-selectin on lung permeability and lung tissue injury. Further cellular or animal experiments are required in the future to clarify this issue.

In summary, the use of immunosuppressive drugs and Klebsiella pneumoniae infection upregulate the expression of p-selectin on platelets. The p-selectin on platelets mediates the aggregation of platelets in the lung. Platelets are activated by inflammatory factors, release a large number of active mediators, and significantly increase the permeability of the air-blood barrier. Severe acute lung injury finally occurs with the participation of immune cells. These results may lead to a more in-depth understanding of the mechanism of acute lung injury induced by infection following organ transplantation and provide new ideas for the development of therapeutic treatments against this lethal disease.

## Figures and Tables

**Table 1:** Sequences of primers used for reverse transcription-quantitative polymerase chain reaction.

Gene	Primer	Sequence
P-selectin	Forward Reverse	5'-CTATACCTGCTCCTGCTACCCA-3' 5'-CTGGAGTCGTAGGCAAAGGC-3'

GAPDH	Forward Reverse	5'-CCTCGTCCCGTAGACAAAATG-3' 5'-TGAGGTCAATGAAGGGGTCGT-3'

## Data Availability

The datasets used and/or analyzed during the present study are available from the corresponding author on reasonable request.
