# Erratum to “The Inhibition of P-Selectin Reduced Severe Acute Lung Injury in Immunocompromised Mice”

**DOI:** 10.1155/2021/9827438

**Published:** 2021-09-28

**Authors:** Yang Liu, Du Xiang, Fang Gao, Hanlin Yao, Qifa Ye, Yanfeng Wang

**Affiliations:** ^1^Zhongnan Hospital of Wuhan University, Institute of Hepatobiliary Diseases of Wuhan University; Transplant Center of Wuhan University, Hubei Key Laboratory of Medical Technology on Transplantation, Wuhan 430071, China; ^2^Binzhou People's Hospital Health Management Center, Binzhou 256600, Shandong Province, China; ^3^Research Center of National Health Ministry on Transplantation Medicine Engineering and Technology, The 3rd Xiangya Hospital of Central South University, Changsha 410000, China

In the article titled “The Inhibition of P-Selectin Reduced Severe Acute Lung Injury in Immunocompromised Mice” [1], the incorrect file for Figures 6(a) and 6(g) was used during the production process and the figure should be corrected as follows:

## Figures and Tables

**Figure 1:**
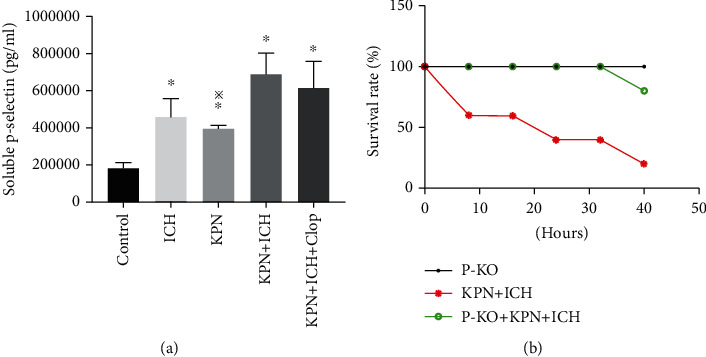
Knockout of p-selectin alleviate acute lung injury in ICH mice. (a) The expression of serum P-selectin in mice and (g) the survival curve of mice.
